# Expression of intramuscular extracellular matrix proteins in vastus lateralis muscle fibres between atrophic and non-atrophic COPD

**DOI:** 10.1183/23120541.00857-2023

**Published:** 2024-05-27

**Authors:** Efpraxia Kritikaki, Gerasimos Terzis, Meera Soundararajan, Ioannis Vogiatzis, Davina C.M. Simoes

**Affiliations:** 1 Faculty of Health and Life Sciences, Northumbria University Newcastle, Newcastle upon Tyne, UK; 2 School of Physical Education and Sports Science, National and Kapodistrian University of Athens, Athens, Greece

## Abstract

**Background:**

Extracellular matrix (ECM) proteins are the major constituents of the muscle cell micro-environment, imparting instructive signalling, steering cell behaviour and controlling muscle regeneration. ECM remodelling is among the most affected signalling pathways in COPD and aged muscle. As a fraction of COPD patients present muscle atrophy, we questioned whether ECM composition would be altered in patients with peripheral muscle wasting (atrophic COPD) compared to those without muscle wasting (non-atrophic COPD).

**Methods:**

A set of ECM molecules with known impact on myogenesis were quantified in vastus lateralis muscle biopsies from 29 COPD patients (forced expiratory volume in 1 s 55±12% predicted) using ELISA and real-time PCR. COPD patients were grouped to atrophic or non-atrophic based on fat-free mass index (<17 or ≥17 kg·m^−2^).

**Results:**

Atrophic COPD patients presented a lower average vastus lateralis muscle fibre cross-sectional area (3872±258 μm^2^) compared to non-atrophic COPD (4509±198 μm^2^). Gene expression of ECM molecules was found significantly lower in atrophic COPD compared to non-atrophic COPD for collagen type I alpha 1 chain (*COL1A1*), fibronectin (*FN1*), tenascin C (*TNC*) and biglycan (*BGN*)*.* In terms of protein levels, there were no significant differences between the two COPD cohorts for any of the ECM molecules tested.

**Conclusions:**

Although atrophic COPD presented decreased contractile muscle tissue, the differences in ECM mRNA expression between atrophic and non-atrophic COPD were not translated at the protein level, potentially indicating an accumulation of long-lived ECM proteins and dysregulated proteostasis, as is typically observed during deconditioning and ageing.

## Shareable abstract

**Intramuscular mRNA expression of crucial extracellular matrix molecules affecting homeostasis is lower in the vastus lateralis muscle of atrophic compared to non-atrophic COPD, suggesting lower baseline transcriptional activity** https://bit.ly/4a2Vdp2

## Introduction

COPD is a progressive lung disease with patients experiencing disabling symptoms despite the use of approved therapies [1]. In COPD, loss of peripheral muscle mass is among the most prevalent extrapulmonary manifestations that constitutes a strong determinant of mortality and is associated with poor clinical outcomes, independent of the degree of airflow obstruction [1, 2]. Besides myofibrillar catabolic protein breakdown, the multinucleated muscle cells lose nuclei, whereas the tissue displays altered adaptive structural features including a diminished proportion of type I fibres, an increased proportion of type II fibres (in particular type IIx), a reduction in muscle fibre cross-sectional area (CSA), impaired oxidative capacity, and lowered mitochondria and capillary density [1]. Multiple aetiologies underlie the loss of peripheral muscle mass in COPD, including muscle disuse, oxidative stress, local inflammation, hypoxaemia, protein anabolism/catabolism imbalance and impaired regenerative capacity [1, 3, 4]. However, the exact mechanisms leading to the underlying peripheral muscle wasting in COPD are not fully understood.

The intramuscular extracellular matrix (ECM) is a highly malleable connective tissue embedding all cells in the muscle and creating the cell micro-environment. The ECM forms a molecular lattice of collagenous components, proteoglycans, matricellular proteins and adhesion receptors which is highly adaptable and responsive to external stimuli, including physical activity and muscle injury [5]. Appreciated for its essential role in force transmission and effective muscle contraction, the ECM also plays a critical role in maintaining muscle homeostasis and mediating muscle adaptation and regeneration [5]. Forming the satellite cell niche, the ECM integrates biophysical and biochemical signals *via* cell–cell and/or cell–matrix communication, essential for muscle regeneration. In ageing and myopathies (*e.g.* Duchenne muscular dystrophy), dysregulated ECM composition compromises muscle function and homeostasis [6]. The ECM degradation and remodelling are among the most affected signalling pathways in COPD and aged muscle [7, 8]. However, the composition of intramuscular ECM has not previously been studied in the peripheral muscles of patients with COPD.

The aim of this retrospective analysis was to investigate the expression of selected intramuscular ECM proteins that are considered to be crucial for muscle homeostasis [5, 9] in the vastus lateralis muscle of COPD patients. Considering that altered intramuscular ECM composition has been found in a number of pathological states and long-term conditions manifesting muscle wasting [6, 7, 10], it was anticipated that ECM expression would differ in the vastus lateralis muscle of COPD patients with muscle wasting (atrophic COPD) compared to those without muscle wasting (non-atrophic COPD).

## Material and methods

### Study population

In the present study, we analysed muscle specimens from 29 male clinically stable COPD patients [11]. COPD patients met the following entry criteria: 1) post-bronchodilator forced expiratory volume in 1 s (FEV_1_) <50% predicted and FEV_1_/forced vital capacity (FVC) <70% without significant post-bronchodilator reversibility (<10% FEV_1_ % pred); and 2) optimal medical therapy without regular use of systemic corticosteroids. A group of 14 young, healthy, sedentary male participants were included [12] to provide a reference for the normal ECM muscle protein composition, as literature is mostly restricted to relative gene expression [5, 9]. This allowed better understanding of changes at the translational level between COPD and healthy individuals. Muscle specimens were analysed at Northumbria University Newcastle (Newcastle upon Tyne, UK) in accordance with the Human Tissue Act 2004 and with approval from Northumbria University Newcastle Ethics Committee (HLSIV220916-V2).

### Muscle biopsy phenotypic analyses

Vastus lateralis muscle percutaneous biopsies were obtained as previously described [11]. Biopsies were analysed blindly for fibre type classification, fibre CSA and capillary/fibre ratio as previously described [11]. The latter analysis refers to the number of capillaries identified in a certain area divided by the number of fibres found in the corresponding muscle section [11].

### Quantitative real-time PCR analysis

Total RNA was extracted using an RNeasy Fibrous Tissue kit (Qiagen) according to the manufacturer's protocol. Quantified with a NanoDrop spectrophotometer (Thermo Fisher Scientific), up to 1 μg RNA was used for synthesising cDNA with SuperScript II reverse transcriptase and RNaseOUT (Invitrogen) [13]. Primers (Eurofins MWG) were designed using Primer3 software (https://primer3.ut.ee). Details on the primers used in this study can be found in the supplementary material. Quantitative real-time PCR data are presented as fold change relative to the housekeeping gene glyceraldehyde 3-phosphate dehydrogenase (*GAPDH*), estimated using the 2^−ΔΔ*C*_t_^ method. Gene expression of the following ECM molecules was analysed: collagen type I heterodimer chains (*COL1A1* and *COL1A2*), collagen type IV (*COL4A1*), fibronectin (*FN1*), tenascin C (*TNC*), integrin β1 (*ITGB1*), osteopontin (*SPP1*), secreted protein acidic and rich in cysteine (*SPARC*), decorin (*DCN*) and biglycan (*BGN*). To gain insight as to whether the changes in ECM in COPD muscle are indicative of the myogenic potential, the expression of two myogenic regulatory factors (MRFs), paired box 7 (*PAX7*) and myogenic differentiation 1 (*MYOD1*), was examined [14].

### Protein extraction and analyses

Muscle tissue was lysed in cOmplete Mini protease inhibitor cocktail (Roche). Total protein was quantified using the Pierce BCA protein assay kit (Thermo Fisher Scientific). Total protein loading onto ELISA was normalised as 1 μg to quantify: collagen I (ab285250; Abcam); collagen IV (orb562147) and integrin β1 (orb563558) (Biorbyt); osteopontin (DOST00), fibronectin (DFBN10) and SPARC (DSP00) (R&D Systems); and tenascin C (EH446RB), decorin (EHDCN) and biglycan (EH45RB) (Invitrogen) according to the manufacturer's instruction. The ELISA's sensitivity and detection range are shown in the supplementary material.

### Statistical analyses

Data on participant demographics and muscle fibre morphology are presented as mean±sem. Differences across the three groups (healthy, atrophic and non-atrophic COPD) were assessed by one-way ANOVA, and between two groups (atrophic and non-atrophic COPD) were assessed by the unpaired t-test. Molecular data presenting a non-normalised distribution were analysed using an unpaired two-tailed Mann–Whitney test. Data are presented as median (25–75% percentiles (interquartile range)). Correlations between the expression level of target genes/proteins and phenotypic characteristics were explored using Spearman's correlation coefficient (r_s_); 95% confidence intervals and significance values (p-values) were calculated. All statistical tests were carried out using Prism version 9.02 (GraphPad, San Diego, CA, USA). The level of statistical significance was set at p<0.05.

## Results

### Study population

COPD patient demographic and lung function characteristics are detailed in table 1. COPD patients were grouped according to their fat-free mass index (FFMI) as non-atrophic (FFMI >17 kg·m^−2^, n=19) and atrophic (FFMI <17 kg·m^−2^, n=10) [4]. The two groups of COPD patients (atrophic and non-atrophic) did not differ in terms of age and severity of airflow obstruction (table 1). Demographics of healthy participants are presented in supplementary table S1.

**TABLE 1:** Demographic and lung function characteristics of non-atrophic and atrophic COPD patients

	COPD	
	Non-atrophic (n=19)	Atrophic (n=10)
**Age (years)**	67.10±1.89	63.00±2.13
**Weight (kg)**	77.35±3.09	62.94±2.29*
**BMI (kg·m^−2^)**	28.81±1.17	21.50±0.66*
**FFMI (kg·m^−2^)**	19.04±0.40	15.64±0.38*
**FEV_1_ (L)**	1.18±0.12	0.98±0.14
**FEV_1_ (% pred)**	44.07±4.84	37.52±6.13
**FVC (L)**	2.84±0.19	2.49±0.17
**FVC (% pred)**	80.55±5.04	72.86±5.94

Data are presented as mean±sem. BMI: body mass index; FFMI: fat-free mass index; FEV_1_: forced expiratory volume in 1 s; FVC: forced vital capacity. *: p<0.05 significance level between non-atrophic and atrophic COPD groups.

Atrophic COPD patients presented a lower average muscle fibre CSA consistent with lower type IIa and type IIx muscle fibre CSA compared to non-atrophic COPD (table 2). Furthermore, COPD patients presented smaller mean fibre CSA, higher distribution of fibre type IIa and lower capillary/fibre ratio compared to healthy individuals (supplementary table S2).

**TABLE 2:** Muscle fibre morphological characteristics of non-atrophic and atrophic COPD patients

	COPD	
	Non-atrophic (n=19)	Atrophic (n=10)
**Fibre type distribution (%)**		
Type I	32.0±3.2	33.6±2.9
Type II	67.5±3.3	65.9±3.0
Type IIa	52.3±3.7	50.4±6.5
Type IIx	15.2±2.2	15.5±3.6
**Cross-sectional area (μm^2^)**		
Mean	4509±198	3872±258*
Type I	4716±271	4717±215
Type IIa	4507±247	3695±337*
Type IIx	3649±208	2872±250*
**Capillary/fibre ratio**	1.41±0.13	1.44±0.09

Data are presented as mean±sem. *: p<0.05 significance level between non-atrophic and atrophic COPD groups.

### Differences in ECM composition between atrophic and non-atrophic COPD

**FIGURE 1:**
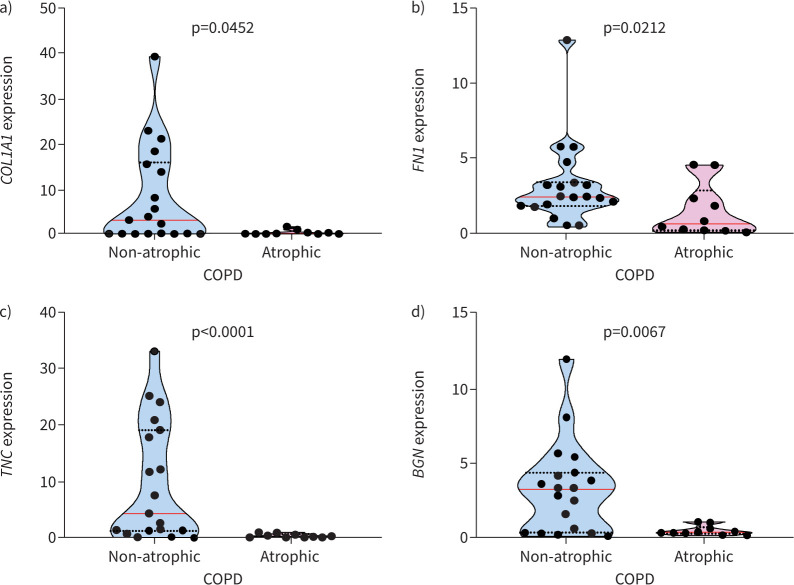
Muscle from atrophic COPD patients shows decreased gene transcription for extracellular matrix proteins compared to non-atrophic. Muscle from atrophic and non-atrophic COPD mRNA expression was quantified for a) collagen type I alpha 1 (*COL1A1*), b) fibronectin (*FN1*), c) tenascin C (*TNC*) and d) biglycan (*BGN*). Violin graphs show the median (red line) and lower and upper quartiles (black dotted lines); individual participant values are represented as black data points. Data are presented as fold change relative to the housekeeping gene glyceraldehyde 3-phosphate dehydrogenase (*GAPDH*).

There were only a few differences in the composition of ECM between atrophic and non-atrophic COPD cohorts. These were limited to changes in mRNA expression levels for *COL1A1*, *FN1*, *TNC* and *BGN*, which were found to be significantly greater in non-atrophic COPD compared to atrophic COPD (figure 1). However, no other differences in mRNA expression were observed between atrophic and non-atrophic COPD patients. In terms of protein levels, there were no significant differences between the two COPD cohorts for all biomarkers tested (table 3).

**TABLE 3:** Protein expression^#^ of extracellular matrix molecules in the vastus lateralis muscle of non-atrophic and atrophic COPD patients

	COPD	
	Non-atrophic	Atrophic
**Collagen type I (pg·mL^−1^)**	293.3 (185.3–443.1)	382.5 (311.9–532.7)
**Collagen type IV (pg·mL^−1^)**	366.3 (247.4–515.3)	467.9 (289.9–586.9)
**Fibronectin (pg·mL^−1^)**	11 950 (6213–17 380)	8847 (7425–9856)
**Integrin β1 (pg·mL^−1^)**	48.4 (35.2–99.9)	62.9 (50.2–94.4)
**Osteopontin (pg·mL^−1^)**	75.68 (44.68–106.6)	62.32 (28.46–315.5)
**Tenascin C (pg·mL^−1^)**	11.6 (5.1–15.2)	9.9 (5.7–12.7)
**SPARC (pg·mL^−1^)**	1504.0 (1128.0–1732.0)	1726.0 (1404.0–1926.0)
**Decorin (pg·mL^−1^)**	44.4 (38.6–51.6)	42.8 (39.5–47.1)
**Biglycan (pg·mL^−1^)**	230.8 (125.7–325.0)	241.3 (170.9–401.9)

Data are presented as median (interquartile range). SPARC: secreted protein acidic and rich in cysteine. ^#^: protein expression was measured with ELISA.

### Myogenic potential is associated with ECM expression

**FIGURE 2:**
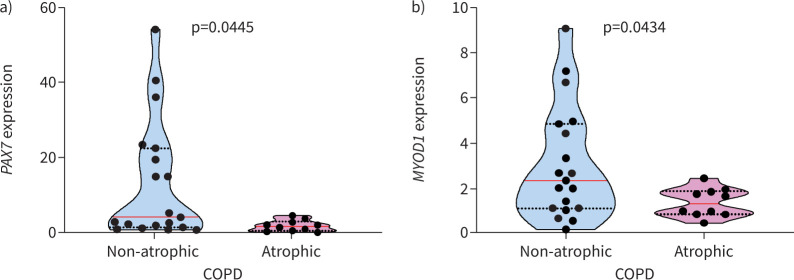
Atrophic COPD patients demonstrate lower paired box 7 (*PAX7*) and myogenic differentiation 1 (*MYOD1*) muscle mRNA expression. a) *PAX7* and b) *MYOD1* mRNA gene expression was analysed in non-atrophic and atrophic COPD patients. Violin graphs show the median (red line) and lower and upper quartiles (black dotted lines); individual participant values are represented as black data points. Data are presented as fold change relative to the housekeeping gene glyceraldehyde 3-phosphate dehydrogenase (*GAPDH*).

Muscle mRNA expression of *PAX7* was significantly greater in non-atrophic COPD compared to atrophic COPD (figure 2a). Similarly, *MYOD1* mRNA was significantly increased in non-atrophic COPD compared to atrophic COPD (figure 2b). Among the ECMs analysed, *SPARC* mRNA expression was positively correlated and associated with the mRNA expression of *PAX7* (r_s_=0.57; p=0.005) and *MYOD1* (r_s_=0.47; p=0.0002) in all COPD patients.

### Structural collagen is decreased in COPD muscle

Differences in muscle ECM composition were observed between COPD and healthy individuals. Specifically, collagen type I protein was significantly lower in individuals with COPD compared to healthy individuals (supplementary table S3). This is consistent with the mRNA expression results, which showed that the collagen type I limiting assembling chain *COL1A2* [15] was significantly lower in COPD patients compared to healthy individuals (supplementary figure S1a). However, no differences were observed in the non-fibrillar collagen type IV protein level (supplementary table S3).

### Adhesion molecules are affected in COPD muscle

Fibronectin is a pro-adhesive glycoprotein, a regulator of myogenesis and a pro-fibrotic marker [5, 16]. In patients with COPD, fibronectin was more abundant compared to healthy controls and was the most abundant protein among the biomarkers measured (table 3 and supplementary table S3). However, at the mRNA level, there was no difference in *FN1* expression between COPD and healthy individuals (supplementary figure S1B). Tenascin C, which inhibits fibronectin-induced cell adhesion migration and ECM remodelling [17], was the least abundant ECM among the biomarkers tested, and it was less abundant in COPD muscle compared to healthy individuals (table 3 and supplementary table S3).

Fibronectin mediates integrin–ECM attachment transmitting signals between ECM and intracellular domains [7, 18, 19]. Among the integrin receptors, integrin β1 is the main receptor for fibronectin. To understand if the increased expression of fibronectin protein in COPD patients leads to an increase in molecular signalling between ECM and intracellular domains, we studied the expression of integrin β1 [18, 19]. However, we found no significant difference in protein and mRNA expression of integrin β1 between COPD and healthy participants (supplementary table S3 and supplementary figure S1c).

### Matricellular ECM proteins accumulate in muscle of COPD patients

COPD is characterised by systemic and local muscle inflammation [1, 20]. The pro-inflammatory osteopontin is a glycoprotein encoded by the *SPP1* gene known to inhibit muscle regeneration in dystrophic and ageing muscle [21, 22]. In COPD patients, osteopontin protein levels were found to be 2.6 times greater compared to healthy individuals (supplementary table S3). However, *SPP1* mRNA expression was found to be downregulated in COPD patients compared to healthy individuals (supplementary figure S1d), indicating that osteopontin protein turnover may be affected.

SPARC, also known as osteonectin, is an exercise-induced myokine which is expressed in various tissues during remodelling/repair and is related to muscle regeneration [23]. SPARC at the protein level was observed to be higher in COPD compared to healthy individuals (supplementary table S3). However, *SPARC* mRNA expression was significantly downregulated in COPD patients compared to healthy individuals (supplementary figure S2a).

### Expression of proteoglycans is altered in COPD

Decorin and biglycan proteoglycans play a significant role in the arrangement of collagen fibrils and myogenesis [9]. The lower level of decorin protein in COPD patients compared to healthy controls (supplementary table S3) may have a negative impact on COPD muscle mass, as decorin binds to myostatin, neutralising its function, and also induces the expression of *MYOD1* and follistatin [5, 24]. Although the mRNA expression of *DCN* was also lower in COPD patients compared to healthy individuals (supplementary figure S2b), the difference was not significant. On the other hand, biglycan, which shares similar glycosaminoglycan chain composition and possibly some of decorin functions [5, 16], was not significantly different between COPD and healthy controls (supplementary table S3 and supplementary figure S2c).

## Discussion

This study presents novel data on the intramuscular expression of crucial ECM molecules affecting homeostasis of the vastus lateralis muscle in COPD patients. The differences in ECM composition between atrophic and non-atrophic COPD were limited to a decrease in mRNA expression of *COL1A1*, *FN1*, *TNC* and *BGN* in atrophic COPD. This suggests that atrophic COPD muscle has lower baseline transcriptional activity, which could be due to severe muscle deconditioning [10, 25]. While atrophic muscle presented decreased contractile muscle tissue, the differences in ECM mRNA expression between atrophic and not atrophic COPD were not translated at the protein level, potentially indicating an accumulation of long-lived ECM proteins and dysregulated proteostasis, as is typically observed during deconditioning and ageing [10, 25]. Accumulation of long-lived ECM proteins in muscle increases the possibility of adverse post-translational modifications known to have adverse effects on aged muscle tissue [26]. According to findings in muscle wasting [10], mRNA expression of ECM molecules was downregulated in COPD muscle, indicating wider gene expression changes. These changes provide insight not only into the effect of COPD on muscle ECM but also into the accelerated ageing process in COPD [27].

The ECM composition in muscles from COPD patients is significantly different from that of healthy young individuals. In COPD muscle, there is an increased presence of glycoproteins such as fibronectin, osteopontin and SPARC, while the abundance of collagen type I, tenascin C and decorin is reduced. These findings are consistent with previous studies showing increased deposition and accumulation of intramuscular ECM in COPD muscle leading to greater levels of fibrosis and replacement of muscle cells with adipose tissue [8, 28].

Type I collagen is the most prevalent type of collagen. Reduced expression of collagen type I in COPD muscle can affect muscle tissue structure, remodelling and force transmission [5, 16]. This reduction in collagen mRNA level is consistent with other studies on ageing and a model of muscle wasting [10]. However, when there are long periods of bed rest or immobilisation, the proportion of collagen and connective tissue in the muscle is reported to be increased, suggesting that inactivity leads to a decline in muscle mass accompanied by accumulation of collagen [29]. Recent studies demonstrated that age-related decline in proteostasis leads to heterogeneity in collagen content and less dense organisation, influencing muscle capacity of adaptation and homeostasis [25]. Additionally, the slowed turnover rate and high half-life of collagens make these proteins subject to the accumulation of deleterious advanced glycation end-products [26].

Matricellular glycoproteins are multifunctional proteins that have the ability to interact with other ECM proteins or cells through integrin receptors [22]. While osteopontin is typically undetectable in normal muscle fibres and increases after muscle injury [30], our research shows that the level of osteopontin protein was 2.6-fold greater in COPD patients compared to healthy individuals. Similarly, in old mice, muscle osteopontin was found to be upregulated by 8-fold in macrophages, 1.5-fold in satellite cells and 2-fold in senescent fibro-adipogenic cells, which was associated with inhibited myogenesis [22, 31]. Although no other studies have explored the regulation of osteopontin in the peripheral muscles of COPD patients, osteopontin plays a critical role in muscle pathology, specifically in Duchenne muscular dystrophy [21]. In fact, inhibition of osteopontin reduces fibrosis and improves muscle function in *mdx* mice [21]. Given that chronic osteopontin overexpression may lead to chronic damage, fibrosis and functional impairment of the damaged muscle [21, 22], the increase in osteopontin levels in COPD muscle compared to the muscle of healthy individuals may be linked with the severity of muscle wasting in COPD.

Fibronectin is an essential glycoprotein for cell adhesion and migration, playing a critical role in muscle regeneration and adaptation [18]. It has been found to increase in muscle from COPD patients and rats after muscle immobilisation [32]. However, its accumulation in the interstitial space can increase the distance for diffusion, making it harder for substrates and hormones to be delivered [18, 19]. Our finding is also in line with a study demonstrating that enhanced abundance in fibronectin levels in the aged gastrocnemius muscle of mice was significantly associated with reduced muscular strength and accompanied by reduced expression of myogenin, a marker of myogenic regeneration and differentiation [33]. Along with fibronectin, tenascin C is critical for ECM remodelling initiation following unloading because it promotes satellite cell expansion during muscle regeneration [9, 16]. However, tenascin C protein levels were found to be lower in COPD patients compared to healthy individuals, which is in agreement with the low levels of tenascin C seen in non-actively remodelling muscle [16, 34]. However, at the mRNA level, *TNC* expression was not homogeneously increased in COPD muscle compared to healthy individuals, confirming earlier findings [8]. Low levels of tenascin C in COPD muscle may affect particularly glycolytic-type muscle fibres as its deficiency in mice leads to fast-twitch muscle fibre mass loss and atrophy [34]. Thus, the dysregulated expression of tenascin C in COPD muscle could mediate loss of muscle mass in these patients. Tenascin C is part of a pleiotropic pathway that protects type II fibre mass from impaired damage-repair cycles in the muscle [35].

SPARC is a protein that has several functions, including protecting and stabilising the cytoskeleton during myogenesis. It is also important for maintaining muscular function [23]. Normally, SPARC facilitates collagen trafficking from areas where it is produced to areas where it is not. However, when SPARC is overexpressed this process is disrupted, and collagen deposition may decrease [23]. In people with COPD, SPARC levels are increased and SPARC accumulates in the serum [36]. To compensate for this, the mRNA expression of *SPARC* decreases in COPD muscle. Calcium influx, which occurs during satellite cell differentiation, controls *SPARC* mRNA expression [37]. This process relies on expression of MRFs *PAX7* and *MYOD1*. Accordingly, the mRNA expression of MRFs *PAX7* and *MYOD1* was associated with *SPARC* [38].

The matrisome, which is a network of proteins that provide structural support to cells, is maintained by interactions of proteoglycans with collagens. In COPD patients, decreased levels of the proteoglycan decorin, along with increased expression of myostatin expression, could negatively impact normal muscle regeneration and growth, potentially by dysregulating the interplay between decorin/myostatin [11, 39]. Myostatin is a well-established inhibitor of muscle mass accretion and a negative regulator of myogenesis, and was found to be greater in COPD patients with muscle wasting [11]. Decorin, on the other hand, can suppress myostatin's activity either by directly binding to the molecule or by indirectly affecting the activity of transforming growth factor-β1 and follistatin. The dysregulated expression of these molecules in the vastus lateralis muscle may partly be responsible for the differences in mean fibre CSA between patients with COPD and healthy individuals.

Considering that most of the studies in muscle ECM are performed in mice tissue, this is a unique study reporting gene expression and protein profiling entirely in human samples and relevant to COPD patients. Certainly, the use of muscle samples from young instead of age-matched healthy individuals is a limiting factor in this study. While the selected ECM molecules studied were not exhaustive, our findings provide a novel conceptual framework for the ECM contribution to muscle adaptation during disuse and muscle wasting in COPD patients. The ECM undergoes remodelling during homeostatic conditions and muscle injury to ensure regular cellular renewal and to support muscle repair and maintenance [5].

As myogenic benefits of regular physical activity are dependent on the ECM micro-environment affecting how cells respond to mechanostimulus [9], our findings may reflect the lower habitual physical activity levels previously reported in patients with COPD compared to healthy individuals [40]. Future studies should investigate whether the reported differences in the ECM profile between atrophic and non-atrophic COPD patients could be altered after an exercise-based rehabilitation programme.

## Supplementary material

10.1183/23120541.00857-2023.Supp1 **Please note:** supplementary material is not edited by the Editorial Office, and is uploaded as it has been supplied by the author.
Supplementary methods 00857-2023.SUPPLEMENT
Figure S1 00857-2023.SUPPLEMENT
Figure S2 00857-2023.SUPPLEMENT2

## References

[1] Maltais F, Decramer M, Casaburi R, et al. An official American Thoracic Society/European Respiratory Society statement: update on limb muscle dysfunction in chronic obstructive pulmonary disease. Am J Respir Crit Care Med 2014; 189: e15–e62. doi:10.1164/rccm.201402-0373ST 24787074 PMC4098112

[2] Attaway AH, Welch N, Hatipoglu U, et al. Muscle loss contributes to higher morbidity and mortality in COPD: an analysis of national trends. Respirology 2021; 26: 62–71. doi:10.1111/resp.13877 32542761

[3] Cao M, Calmelat RA, Kierstead P, et al. A randomized, crossover, placebo controlled, double-blind trial of the effects of tiotropium-olodaterol on neuromuscular performance during exercise in COPD. J Appl Physiol 2022; 132: 1145–1153. doi:10.1152/japplphysiol.00332.2021 35323052 PMC9054255

[4] Wagner PD. Possible mechanisms underlying the development of cachexia in COPD. Eur Respir J 2008; 31: 492–501. doi:10.1183/09031936.00074807 18310396

[5] Mavropalias G, Boppart M, Usher KM, et al. Exercise builds the scaffold of life: muscle extracellular matrix biomarker responses to physical activity, inactivity, and aging. Biol Rev Camb Philos Soc 2023; 98: 481–519. doi:10.1111/brv.12916 36412213

[6] Pavan P, Monti E, Bondi M, et al. Alterations of extracellular matrix mechanical properties contribute to age-related functional impairment of human skeletal muscles. Int J Mol Sci 2020; 21: 3992. doi:10.3390/ijms21113992 32498422 PMC7312402

[7] Schuler SC, Kirkpatrick JM, Schmidt M, et al. Extensive remodeling of the extracellular matrix during aging contributes to age-dependent impairments of muscle stem cell functionality. Cell Rep 2021; 35: 109223. doi:10.1016/j.celrep.2021.109223 34107247

[8] Willis-Owen SAG, Thompson A, Kemp PR, et al. COPD is accompanied by co-ordinated transcriptional perturbation in the quadriceps affecting the mitochondria and extracellular matrix. Sci Rep 2018; 8: 12165. doi:10.1038/s41598-018-29789-6 30111857 PMC6093887

[9] Kritikaki E, Asterling R, Ward L, et al. Exercise training-induced extracellular matrix protein adaptation in locomotor muscles: a systematic review. Cells 2021; 10: 1022. doi:10.3390/cells10051022 33926070 PMC8146973

[10] Guilhot C, Fovet T, Delobel P, et al. Severe muscle deconditioning triggers early extracellular matrix remodeling and resident stem cell differentiation into adipocytes in healthy men. Int J Mol Sci 2022; 23: 5489. doi:10.3390/ijms23105489 35628300 PMC9143135

[11] Vogiatzis I, Simoes DCM, Stratakos G, et al. Effect of pulmonary rehabilitation on muscle remodelling in cachectic patients with COPD. Eur Respir J 2010; 36: 301–310. doi:10.1183/09031936.00112909 20110400

[12] Tsitkanou S, Spengos K, Stasinaki AN, et al. Effects of high-intensity interval cycling performed after resistance training on muscle strength and hypertrophy. Scand J Med Sci Sports 2017; 27: 1317–1327. doi:10.1111/sms.12751 27659479

[13] Simoes DCM, Paschalidis N, Kourepini E, et al. An integrin axis induces IFN-beta production in plasmacytoid dendritic cells. J Cell Biol 2022; 221: e202102055. doi:10.1083/jcb.202102055 35878016 PMC9354318

[14] Zammit PS. Function of the myogenic regulatory factors Myf5, MyoD, myogenin and MRF4 in skeletal muscle, satellite cells and regenerative myogenesis. Semin Cell Dev Biol 2017; 72: 19–32. doi:10.1016/j.semcdb.2017.11.011 29127046

[15] Ricard-Blum S. The collagen family. Cold Spring Harb Perspect Biol 2011; 3: a004978. doi:10.1101/cshperspect.a004978 21421911 PMC3003457

[16] Csapo R, Gumpenberger M, Wessner B. Skeletal muscle extracellular matrix – what do we know about its composition, regulation, and physiological roles? A narrative review. Front Physiol 2020; 11: 253. doi:10.3389/fphys.2020.00253 32265741 PMC7096581

[17] Midwood KS, Chiquet M, Tucker RP, et al. Tenascin-C at a glance. J Cell Sci 2016; 129: 4321–4327. 27875272 10.1242/jcs.190546

[18] Lukjanenko L, Jung MJ, Hegde N, et al. Loss of fibronectin from the aged stem cell niche affects the regenerative capacity of skeletal muscle in mice. Nat Med 2016; 22: 897–905. doi:10.1038/nm.4126 27376579 PMC5467443

[19] Rozo M, Li L, Fan CM. Targeting beta1-integrin signaling enhances regeneration in aged and dystrophic muscle in mice. Nat Med 2016; 22: 889–896. doi:10.1038/nm.4116 27376575 PMC4974124

[20] Vogiatzis I, Terzis G, Nanas S, et al. Skeletal muscle adaptations to interval training in patients with advanced COPD. Chest 2005; 128: 3838–3845. doi:10.1378/chest.128.6.3838 16354852

[21] Capote J, Kramerova I, Martinez L, et al. Osteopontin ablation ameliorates muscular dystrophy by shifting macrophages to a pro-regenerative phenotype. J Cell Biol 2016; 213: 275–288. doi:10.1083/jcb.201510086 27091452 PMC5084275

[22] Paliwal P, Pishesha N, Wijaya D, et al. Age dependent increase in the levels of osteopontin inhibits skeletal muscle regeneration. Aging 2012; 4: 553–566. doi:10.18632/aging.100477 22915705 PMC3461343

[23] Jorgensen LH, Jepsen PL, Boysen A, et al. SPARC interacts with actin in skeletal muscle *in vitro* and *in vivo*. Am J Pathol 2017; 187: 457–474. doi:10.1016/j.ajpath.2016.10.013 27908613

[24] Miura T, Kishioka Y, Wakamatsu J, et al. Interaction between myostatin and extracellular matrix components. Anim Sci J 2010; 81: 102–107. doi:10.1111/j.1740-0929.2009.00700.x 20163680

[25] Abbott CB, Lawrence MM, Kobak KA, et al. A novel stable isotope approach demonstrates surprising degree of age-related decline in skeletal muscle collagen proteostasis. Function 2021; 2: zqab028. doi:10.1093/function/zqab028 34124684 PMC8187230

[26] Suzuki A, Yabu A, Nakamura H. Advanced glycation end products in musculoskeletal system and disorders. Methods 2022; 203: 179–186. doi:10.1016/j.ymeth.2020.09.012 32987130

[27] Lakhdar R, McGuinness D, Drost EM, et al. Role of accelerated aging in limb muscle wasting of patients with COPD. Int J Chron Obstruct Pulmon Dis 2018; 13: 1987–1998. doi:10.2147/COPD.S155952 29970961 PMC6022820

[28] Gosker HR, Kubat B, Schaart G, et al. Myopathological features in skeletal muscle of patients with chronic obstructive pulmonary disease. Eur Respir J 2003; 22: 280–285. doi:10.1183/09031936.03.00012803 12952261

[29] Thot GK, Berwanger C, Mulder E, et al. Effects of long-term immobilisation on endomysium of the soleus muscle in humans. Exp Physiol 2021; 106: 2038–2045. doi:10.1113/EP089734 34387385

[30] Wasgewatte Wijesinghe DK, Mackie EJ, Pagel CN. Normal inflammation and regeneration of muscle following injury require osteopontin from both muscle and non-muscle cells. Skelet Muscle 2019; 9: 6. doi:10.1186/s13395-019-0190-5 30808406 PMC6390361

[31] Zhang X, Habiballa L, Aversa Z, et al. Characterization of cellular senescence in aging skeletal muscle. Nat Aging 2022; 2: 601–615. doi:10.1038/s43587-022-00250-8 36147777 PMC9491365

[32] Salonen V, Lehto M, Kalimo M, et al. Changes in intramuscular collagen and fibronectin in denervation atrophy. Muscle Nerve 1985; 8: 125–131. doi:10.1002/mus.880080208 3903490

[33] Sosa P, Alcalde-Estévez E, Asenjo-Bueno A, et al. Aging-related hyperphosphatemia impairs myogenic differentiation and enhances fibrosis in skeletal muscle. J Cachexia Sarcopenia Muscle 2021; 12: 1266–1279. doi:10.1002/jcsm.12750 34337906 PMC8517361

[34] Zhou S, Zhang W, Cai G, et al. Myofiber necroptosis promotes muscle stem cell proliferation via releasing Tenascin-C during regeneration. Cell Res 2020; 30: 1063–1077. doi:10.1038/s41422-020-00393-6 32839552 PMC7784988

[35] Fluck M, Mund SI, Schittny JC, et al. Mechano-regulated tenascin-C orchestrates muscle repair. Proc Natl Acad Sci USA 2008; 105: 13662–13667. doi:10.1073/pnas.0805365105 18757758 PMC2533246

[36] Qaisar R, Karim A, Muhammad T, et al. Prediction of sarcopenia using a battery of circulating biomarkers. Sci Rep 2021; 11: 8632. doi:10.1038/s41598-021-87974-6 33883602 PMC8060253

[37] Cho WJ, Kim EJ, Lee SJ, et al. Involvement of SPARC in *in vitro* differentiation of skeletal myoblasts. Biochem Biophys Res Commun 2000; 271: 630–634. doi:10.1006/bbrc.2000.2682 10814513

[38] Petersson SJ, Jorgensen LH, Andersen DC, et al. SPARC is up-regulated during skeletal muscle regeneration and inhibits myoblast differentiation. Histol Histopathol 2013; 28: 1451–1460. doi:10.14670/HH-28.1451 23670848

[39] Kneppers AEM, Langen RCJ, Gosker HR, et al. Increased myogenic and protein turnover signaling in skeletal muscle of chronic obstructive pulmonary disease patients with sarcopenia. J Am Med Dir Assoc 2017; 18: 637.e631–637.e611. doi:10.1016/j.jamda.2017.04.016 28578881

[40] Burtin C, Mohan D, Troosters T, et al. Objectively measured physical activity as a COPD clinical trial outcome. Chest 2021; 160: 2080–2100. doi:10.1016/j.chest.2021.06.044 34217679

