# Supplementary material for: Expression of intramuscular extracellular matrix proteins in vastus lateralis muscle fibres between atrophic and non-atrophic COPD
Source: ERJ Open Res. 2024 May 27;10(3):00857-2023. doi: 10.1183/23120541.00857-2023 (PMC11129643; doi:10.1183/23120541.00857-2023)
Supplement: Supplementary file 1 [file 00857-2023.SUPPLEMENT.pdf]

**ONLINE SUPPLEMENT**

**MATERIAL AND METHODS:**

**Primer sequences used in this study:**

*COL1A1* sense, 5'-CACGACAAAGCAGAAACAT-3', antisense 5'-TTCAGCAACACAGTTACAC-3'; *COL1A2* sense, 5'-GGATGAGGAGACTGGCAAC-3', antisense 5'-GTGATGGCTTATTTGTTTTG-3'; *FN1* sense 5'-GAAAGACCAGCAGAGGCATA-3', antisense 5'-ACTCATCTCCAACGGCATAA-3'; *TNC* sense 5'- ACCCACTACACAGCCAAGA-3', antisense 5'- TCAGGAACACAATCCATCCA; *SPP1* sense 5'- CTACCTTCGCCTATGTTGTCA-3', antisense 5'-GTGATTAGTGATGTTGAGCA-3'; *SPARC* sense 5'- TGAGAGGGATGAGGACAACAA-3', antisense 5'- ATGAGGGGAGCACGCAGT-3'; *DCN*: sense 5'-TTGGAACGACTTTATCTGTCC-3', antisense 5'-TAAGGGAAGGAGGAAGACCT-3'; *BGN*: sense 5'-CCTGGGTCTGAAGTCTGT-3', antisense 5'-GTGGTTCTTGGAGATGTAGA-3'; *ITGB1*: sense 5'-CCTTTTTGACCTTTTCTTCCTG-3', antisense 5'-GACATTTACTTTGGATTCT-3'; *PAX7*: sense 5'-CAGTCAACAAGACATTCCTA-3', antisense 5'-TGAAGATGAGAAACAGGTA-3'; *MYOD1*: sense 5'-AGACCCTCGCAGACCTAA-3', antisense 5'-CTGGCAAAGCAACTCTTAT-3'; *Myostatin*: sense 5'-ACCATGCCTACAGAGTCTGA-3', antisense 5'-GATTCAGGTTGTTTGAGCCAA-3'. Real-time PCR was performed with SYBRGreen I (Molecular Probes) and Platinum Taq DNA polymerase (Invitrogen) in a StepOnePlus RT-PCR system (AppliedBiosystems). Analysis was performed using the $2^{-\Delta\Delta Ct}$ method [1]. All values were normalized against expression of the glyceraldehyde-3-phosphate dehydrogenase (*GAPDH*: sense 5'-GAAGGTGAAGGTCGGAGT-3', antisense 5'-CATGGGTGGAATCATATTGGAA-3') [2].

**Protein analyses using ELISA.**

Proteins were analysed using ELISA, which sensitivity and detection range are described for the following kits: collagen I (ab285250, Abcam, 0.188 ng/ml, 0.31 – 20 ng/ml); collagen IV (orb562147, 0.188 ng/ml, 0.31 – 20 ng/ml) and integrin beta 1 (orb563558, 9.375 pg/ml, 15.6 – 1000 pg/ml) from Biorbyt; osteopontin (DOST00, 0.024 ng/ml, 0.3 – 20 ng/ml), fibronectin (DFBN10, 0.579 ng/ml, 3.1 – 200 ng/ml), SPARC (DSP00, 0.269 ng/ml, 1.6 – 50 ng/ml) from R&D Systems; tenascin C (EH446RB, 6.1 pg/ml, 6.1 – 1500 pg/ml), decorin (EHDCN, 1.50 pg/ml, 0.96 – 700 pg/ml), and biglycan (EH45RB, 0.51 ng/ml, 0.51 – 125 ng/ml) from Thermo Fisher Scientific. Acquired standard curves for individual ELISA kits are shown in Supplementary Figure 3.

**Supplementary Table 1. Demographic characteristics of healthy individuals and COPD patients**

| Characteristics | COPD | Healthy |
|---|---|---|
| **Individuals (n)** | 29 | 14 |
| **Age (years)** | 65.68 ± 1.42* | 21.80 ± 0.60 |
| **Weight (kg)** | 72.89 ± 2.62 | 74.20 ± 2.10 |
| **BMI ($kg \bullet m^{-2}$)** | 25.94 ± 0.80 | 23.45 ± 0.58 |
| **FFM index ($kg \bullet m^{-2}$)** | 17.76 ± 0.40 | 23.70 ± 0.67 |
| **$FEV_1$ (L)** | 1.13 ± 0.08 | – |
| **$FEV_1$ (% predicted)** | 43.32 ± 3.29 | – |
| **FVC (L)** | 2.73 ± 0.14 | – |
| **FVC (% predicted)** | 78.80 ± 3.91 | – |

Data are presented as mean ± SEM. BMI: body mass index; FFM: fat-free mass; $FEV_1$: forced expiratory volume in the first second; FVC: forced vital capacity; Asterisks indicate $p$<0.05 significance level between COPD and healthy groups.

**Supplementary Table 2. Muscle fibre morphological characteristics of healthy individuals and COPD patients.**

| Muscle fibre characteristics | COPD | Healthy |
|---|---|---|
| **Individuals (n)** | 29 | 14 |
| **Fibre type distribution (%)** | | |
| **Type I** | 33.0 ± 2.9 | 39.8 ± 4.6 |
| **Type II** | 65.9 ± 2.4 | 59.2 ± 5.9 |
| **Type IIa** | 52.0 ± 1.9* | 38.9 ± 2.8 |
| **Type IIx** | 15.5 ± 0.7 | 20.2 ± 3.1 |
| **Cross sectional area (CSA, μm$^2$)** | | |
| **Mean CSA** | 4100 ± 106.9* | 4582 ± 155.6 |
| **Type I** | 4763 ± 171.9* | 3974 ± 146.8 |
| **Type IIa** | 4154 ± 138.7* | 5656 ± 205.8 |
| **Type IIx** | 3339 ± 165.2* | 4176 ± 180.2 |
| **Capillary/fibre ratio** | 1.43 ± 0.07* | 2.02 ± 0.56 |

Data are presented as mean ± SEM. Asterisks indicate *p*<0.05 significance level between COPD and healthy control group.

**Supplementary Table 3. Protein expression of ECM molecules in vastus lateralis muscle of COPD patients and healthy controls.**

| Protein (pg/ml) | COPD | Healthy | p-value |
|---|---|---|---|
| **COL1** | 380.5* (256.0-457.4) | 6441 (1423-11580) | **<0.0001** |
| **COL4** | 372.3 (281.1-530.8) | 416.9 (309.0-624.7) | 0.3621 |
| **Fibronectin** | 9418.0* (6271.0-13150.0) | 3918.0 (2867.0-5972.0) | **0.0002** |
| **ITGβ1** | 55.9 (40.8-96.9) | 61.6 (49.9-73.5) | 0.4852 |
| **Osteopontin** | 70.34* (30.60-146.9) | 26.78 (20.65-66.8) | **0.0194** |
| **Tenascin C** | 10.4* (5.1-14.2) | 16.8 (6.1-22.0) | **0.0403** |
| **SPARC** | 1612.0* (1186.0-1880.0) | 1224.0 (940.5-1342.0) | **0.0095** |
| **Decorin** | 42.8* (38.68-48.1) | 71.1 (57.4-85.9) | **<0.0001** |
| **Biglycan** | 230.8 (137.2-376.6) | 169.9 (111.2-250.3) | 0.1430 |

Protein expression was measured with ELISA. Data are shown as median (25 − 75 % percentile). Asterisks indicate $p$<0.05 significance level between COPD and healthy control group.
