# Supplementary figures and images for: Expression of intramuscular extracellular matrix proteins in vastus lateralis muscle fibres between atrophic and non-atrophic COPD
Source: ERJ Open Res. 2024 May 27;10(3):00857-2023. doi: 10.1183/23120541.00857-2023 (PMC11129643; doi:10.1183/23120541.00857-2023)

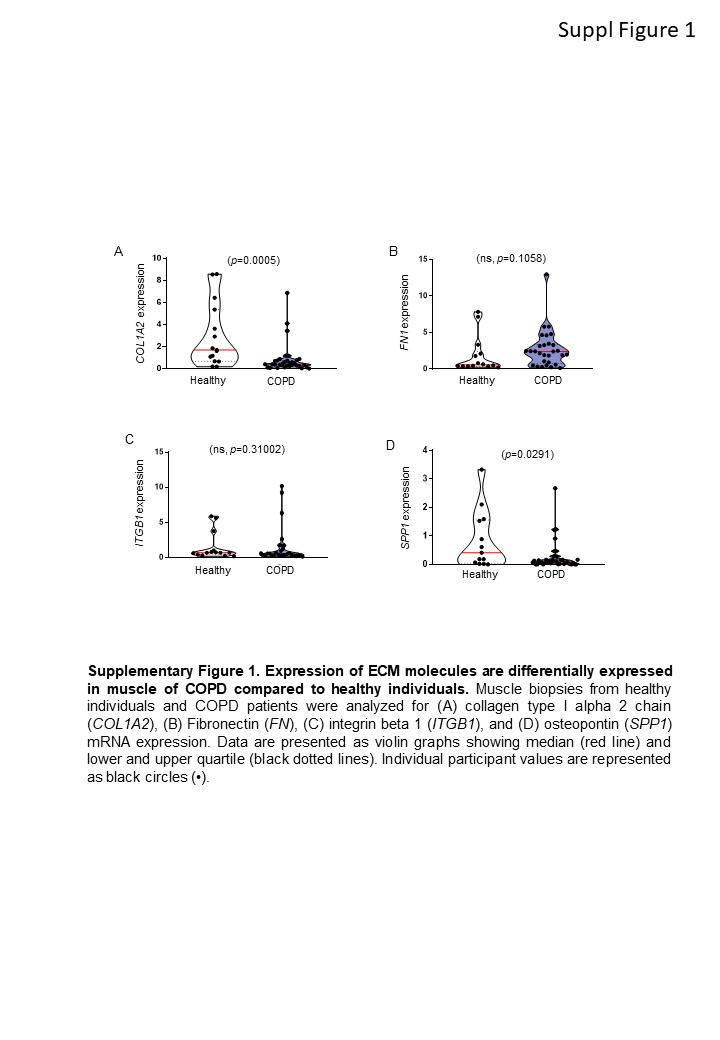

Supplement: Supplementary file 2 [file 00857-2023.SUPPLEMENT.jpg]

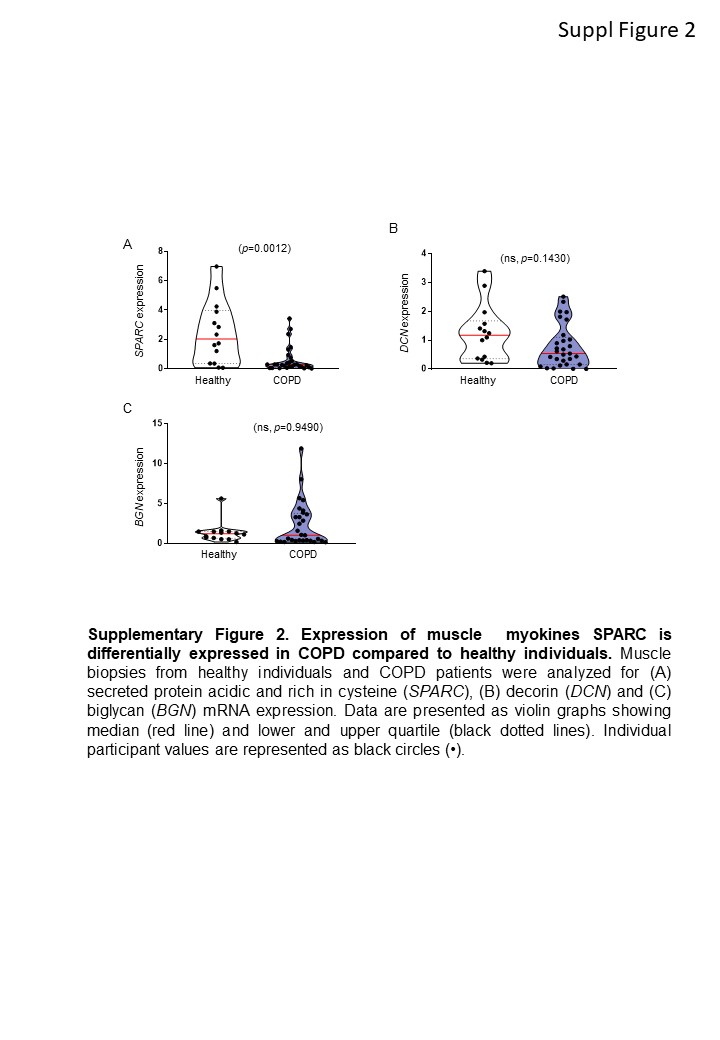

Supplement: Supplementary file 3 [file 00857-2023.SUPPLEMENT2.jpg]
